# Efficacy and Safety of Conjoint Fascial Sheath (CFS) Suspension in the Treatment of Blepharoptosis: A Systematic Review and Meta-analysis

**DOI:** 10.1007/s00266-025-04724-z

**Published:** 2025-02-21

**Authors:** Dongshuo Ji, Ying Liu, Xing Han, Shouduo Hu, Yanyong Zhao

**Affiliations:** 1 Plastic Surgery Department, Beijing Hospital of Integrated Traditional Chinese and Western Medicine, Beijing, 100038 China; 2 Ear Reconstruction Center, Plastic Surgery Hospital, Chinese Academy of Medical Sciences, Peking Union Medical College, Beijing, 100144 China

**Keywords:** Conjoint fascial sheath, Blepharoptosis, Systematic review, Meta-analysis

## Abstract

**Background:**

This study focuses on blepharoptosis, a common condition in oculoplastic surgery characterized by drooping of the upper eyelid. The efficacy and safety of using the combined fascial sheath (CFS) to correct blepharoptosis are still unclear.

**Methods:**

A systematic search encompassing four databases (PubMed, Embase, Web of Science, and Cochrane Library) up to December 15, 2023, was conducted. The meta-analysis was performed using Stata 14.

**Results:**

A total of 10 studies involving 683 patients were included. The meta-analysis indicated a significant improvement in mean marginal reflex distance 1 (MRD1) in the CFS group compared to the control group (WMD: 2.33; 95% CI 1.61 to 3.06; *I*^2^=97.2%, *P* < 0.001). Additionally, patient satisfaction in the CFS group was significantly higher than in the control group (OR: 5.28; 95% CI 1.71 to 16.32; *I*^2^=0, *P* = 0.683), and the complication rate was significantly lower (OR 0.26; 95% CI 0.14 to 0.48; *I*^2^=0, *P* = 0.899). However, no significant difference in curative effect was observed between the two groups (RD: 0.15; 95% CI −0.01 to 0.31; *I*^2^=88.3%, *P* < 0.001).

**Conclusion:**

CFS suspension has the potential to significantly improve MRD1 and patient satisfaction, while exhibiting a low incidence of complications and a favorable safety profile. These findings indicate that clinicians might consider CFS suspension as a viable treatment option for blepharoptosis. However, it is important to exercise caution due to inter-study heterogeneity and the limitations of current analysis.

**Level of Evidence III:**

This journal requires that authors assign a level of evidence to each article. For a full description of these Evidence-Based Medicine ratings, please refer to the Table of Contents or the online Instructions to Authors www.springer.com/00266

**Supplementary Information:**

The online version contains supplementary material available at 10.1007/s00266-025-04724-z.

## Introduction

Within the realm of ocular plastic surgery, blepharoptosis is a common occurrence with multiple inducement mechanisms [1]. These mechanisms encompass congenital ptosis, where low function of fibroadipose tissue in the levator palpebrae superioris (LPS) muscle is implicated, myogenic ptosis arising from dysgenesis-induced weakness of the LPS muscle, and neurogenic ptosis stemming from complete or partial loss of cranial nerve III [2, 3]. Blepharoptosis is characterized by the drooping of one or both sides of the upper eyelid, leading to a narrowed palpebral fissure and obscuring the eyes [4]. Additionally, it may be linked to other eye diseases or systemic conditions [5, 6]. Aponeurosis repair and levator myectomy are favored treatment options for blepharoptosis. In situations of severe ptosis and diminished levator function, frontalis suspension is a widely employed surgical solution [7]. This procedure establishes a connection between the frontalis and tarsus, thereby rectifying the eyelid position using the elevatory force generated by the frontalis muscle [8]. Despite its utility, frontalis suspension does not completely satisfy normal physiological requirements and is commonly associated with postoperative keratitis. Vulnerable patients, in particular, are prone to developing corneal complications [9].

The conjoint fascial sheath (CFS), alternatively referred to as the check ligament, suspensory ligament of the superior fornix, inferior ligament of Whitnall, and transverse superior fascial expansion, constitutes a robust fibrous sheath positioned above the Müller muscle. It has been acclaimed as an optimal fixation point and has proved effective in the correction of open blepharoptosis [10–14]. In 2002, Holmström and Santanelli were the first to apply the CFS for the correction of blepharoptosis [10]. Zhou introduced the technique of minimally invasive CFS suspension in 2019, aiming to treat mild and moderate ptosis [15]. In 2020, Xing and Wang executed a combined suspension of CFS and levator muscle complex for the treatment of severe ptosis [16]. The objective of this systematic review and meta-analysis is to critically evaluate the existing body of evidence regarding the efficacy and safety of CFS suspension in the treatment of blepharoptosis. As the demand for effective and safe ptosis correction procedures continues to rise, a thorough examination of CFS suspension is essential to guide clinicians in decision-making and patient management.

## Materials and Methods

Adhering to the Preferred Reporting Items for Systematic Reviews and Meta-Analyses (PRISMA) statement, this systematic review was carried out [17].

### Source and Search Strategy

A comprehensive search, encompassing PubMed, Embase, Web of Science, and the Cochrane Library, was conducted from their inception until December 15, 2023. The search utilized a combination of keywords, including “blepharoptosis,” “eyelid ptosis,” “drooping eyelid,” “eye ptosis,” and “conjoint fascial sheath.” Additionally, a manual search of reference lists from included articles, relevant meta-analyses, and reviews was performed. Detailed search strategies are provided in Additional file 1 (Table S1). We extended the review to include abstracts from congresses and scientific meetings, reference lists of retrieved articles, published study protocols, previously published systematic reviews, and review articles, with no language restrictions.

### Eligibility Criteria

The inclusion criteria for this study were as follows: (1) population: patients diagnosed with blepharoptosis; (2) intervention: studies that involved CFS as an intervention measure; (3) study design: both randomized controlled trials (RCTs) and cohort studies were included; and (4) outcomes: the primary outcomes assessed in this study were mean MRD1, curative effect, patient satisfaction, and complication rate. Exclusion criteria comprised: (1) case reports or case series patients; (2) non-original studies including reviews and editorials; (3) partially overlapping patient cohorts; (4) articles not written in English; and (5) non-human studies. The screening of the literature was independently performed by two reviewers, and a consensus was reached.

### Data Extraction and Quality Assessment

Using Microsoft Excel software, data extraction onto standardized forms was conducted. The process was carried out by one researcher and independently reviewed by another. Standard tables included information such as the author, release date, study design, patients, sample size, age, percentage of females, intervention in the experimental group, intervention in the controlled group, follow-up duration, arms involved, and outcomes. Quality assessment for randomized controlled trials (RCTs) utilized the Cochrane risk-of-bias assessment tool, considering randomization methods, blinding, allocation concealment, data completeness, publication bias, and other biases. Non-randomized comparative studies (NRCSs) were assessed using the Methodological Index for Non-Randomized Studies (MINORS) [18].

### Statistical Analysis

STATA software package (version 14, STATA Corporation) was used for data management, effect size transformation, and calculation of pooled results. Forest plots were generated to illustrate the effect estimate, 95% confidence interval (CI) for each study, and the weight assigned to each study in the meta-analysis, along with the overall pooled result. Heterogeneity of effect sizes across studies was tested using Cochran’s Q (reported as *χ*2 and *P* values) and the *I*^2^ statistic [19]. The *I*^2^ statistic represents the percentage of variability in effect estimates, independent of the number of studies, with values of 25%, 50%, and 75% indicating low-, moderate-, and high-level heterogeneity, respectively. As heterogeneity was high, random-effects models were used for data pooling [20]. Sensitivity analyses were conducted to assess the influence of each study on the overall pooled results. Subgroup analyses based on the time of follow-up (> 6 months or ≤ 6 months) were performed. Potential publication bias was evaluated through visual inspection of the funnel plot and testing using Begg’s and Egger’s tests [21]. A *P* value < 0.05 was considered statistically significant.

## Results

### Literature Search

**Fig. 1:**
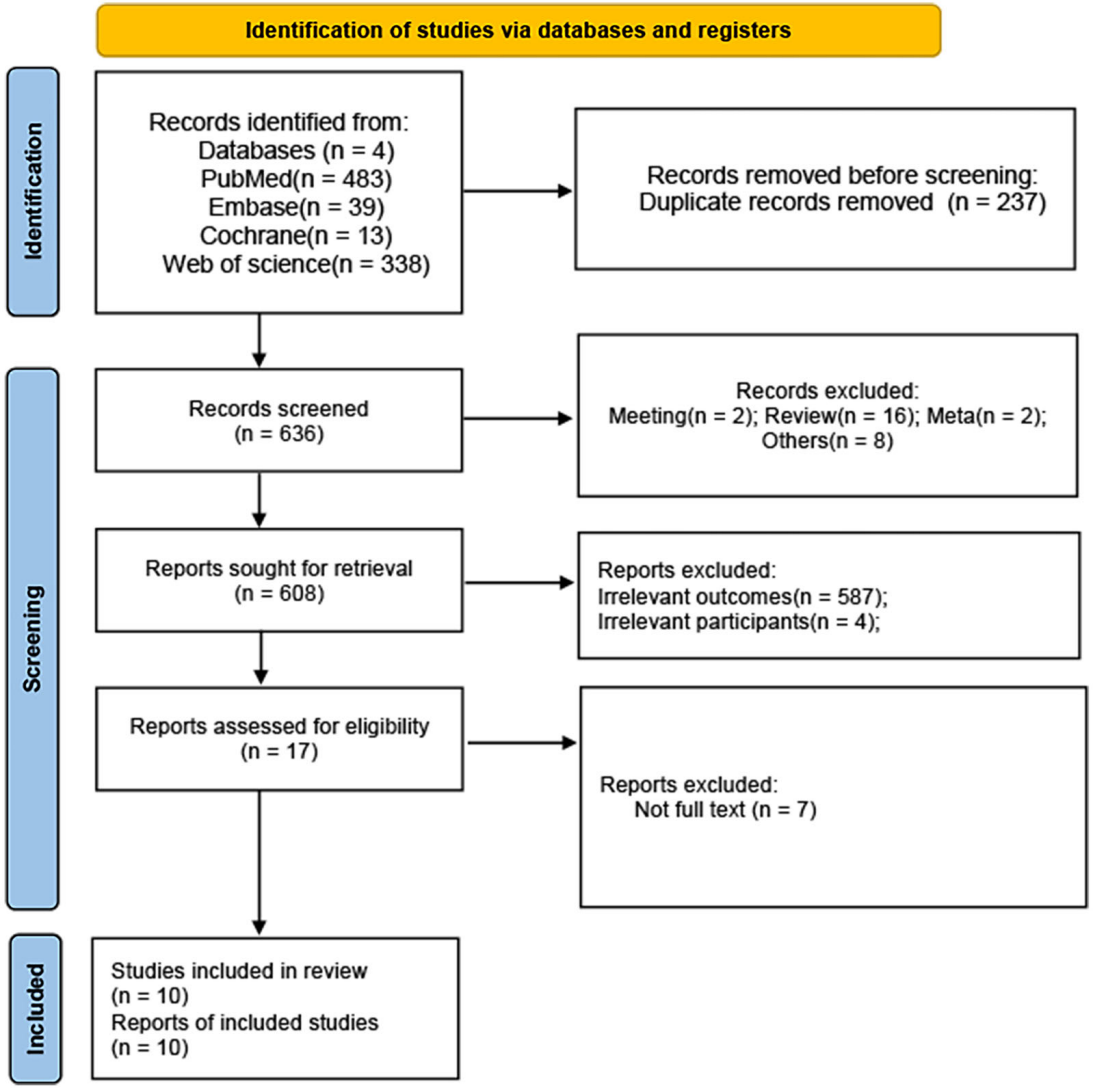
Flowchart of studies’ selection process

**Fig. 2:**
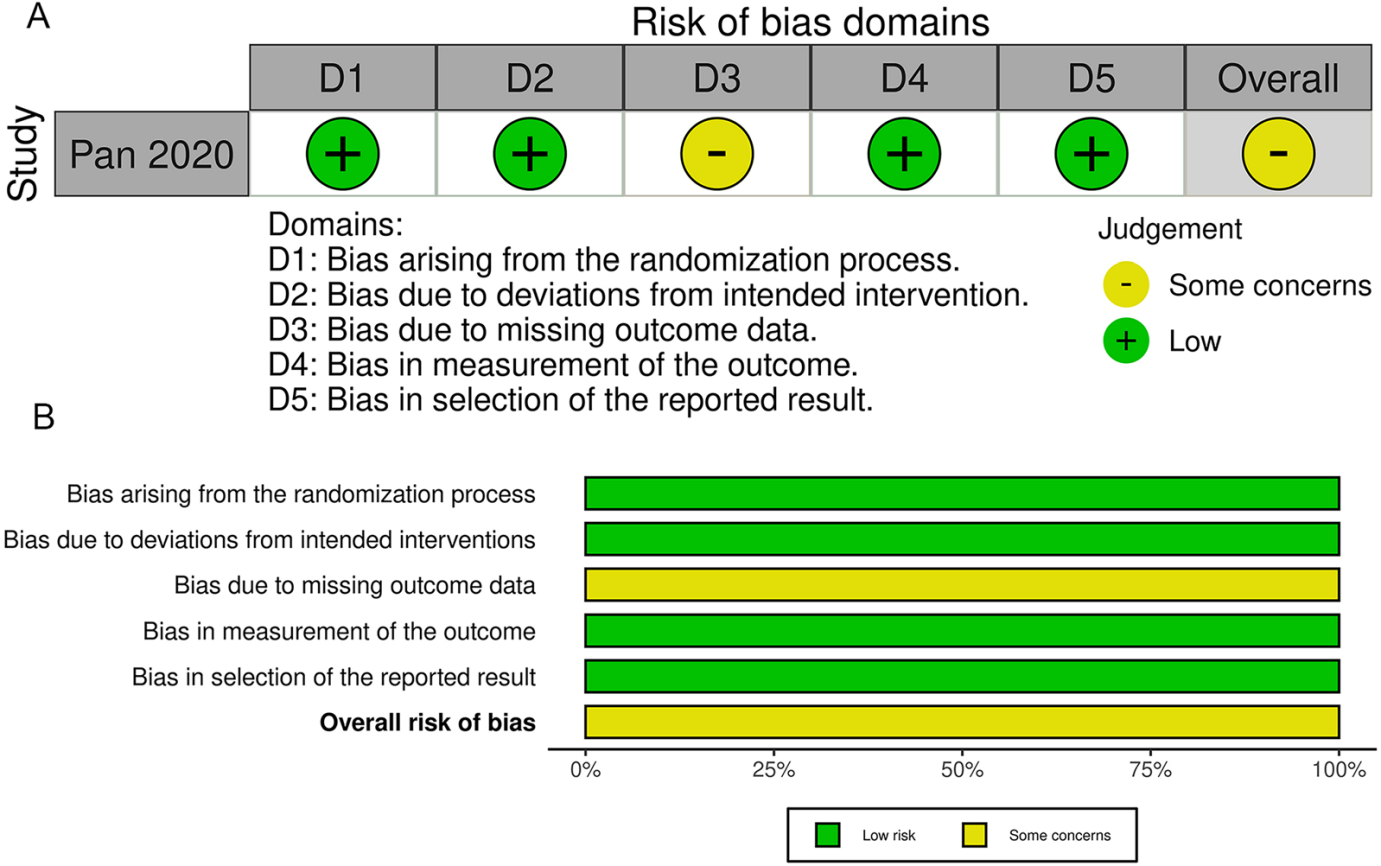
Risk-of-bias assessment in individual RCT analyzed. **A** Risk-of-bias summary, **B** risk-of-bias graph

The search flowchart, as presented in Fig. 1, outlines our exploration process. We systematically searched databases, including PubMed, the Cochrane Library, Web of Science, and Embase, leading to the identification of a total of 873 articles (Fig. 1). Following this, all located papers were imported into EndnoteX9 software, and the exclusion of duplicate papers (*n* = 237) took place. Additionally, 619 papers were excluded based on the review of titles and abstracts. During the initial screening, 17 papers aligned with the study’s topic; however, 7 papers were excluded due to the unavailability of full text. Ultimately, the final analysis comprised 10 studies [16, 22–30], involving a total of 683 patients (Fig. 1). Table 1 provides a comprehensive view of the characteristics of all the studies included in the analysis. It includes information such as the author’s name, publication year, study design, sample size, country, age, percentage of females, intervention in the experimental group, intervention in the controlled group, follow-up duration, arms, and outcomes. Among the 10 studies, 9 were conducted in China, and 1 was conducted in South Korea. There were five double-armed studies, and the remaining studies were single arm. As for included type of ptosis patients, eight of the ten studies focused on patients with severe ptosis. This group included one study on patients with severe involutional ptosis and four studies on those with severe congenital ptosis. The remaining two studies targeted patients with moderate to severe congenital ptosis and those with mild to moderate ptosis, respectively. The studies comprised one RCT and nine cohort studies. Figure 2 displays the quality assessment of the RCT. Quality assessment scores for the nine non-randomized comparative studies (NRCSs) ranged from 16 to 24, evaluated using the MINORS tool (Table S2).

**Table 1:** Characteristics of the studies included in this meta-analysis

Study	Country	Study design	Patients	Sample size (experiment/control)	Age	Female (%)	Comparison	Follow-up	Type of ptosis	Outcomes	Arms
Liu 2021[22]	China	Cohort study	Severe involutional blepharoptosis	67	55	62.69	Postoperative versus preoperative	12 months	Severe involutional ptosis	MRD1, efficacy	Single arm
Shi 2021[23]	China	Cohort study	Congenital severe ptosis	182(89/93)	9.98	48.35	CFS+LM versus. FMF	6 months	Congenital severe ptosis	Efficacy, complication	Double arm
Pan 2020[24]	China	RCT	Moderate or severe congenital ptosis	75(38/37)	21.89	54.67	CFS versus FM	3 months	Moderate or severe congenital ptosis	Efficacy, complication	Double arm
Wang 2022[25]	China	Cohort study	Severe congenital blepharoptosis	50	8.62	24	≤7 years versus >7 years	6 months	Severe congenital blepharoptosis	MRD1, efficacy, complication	Double arm
Wang 2020[26]	China	Cohort study	Severe blepharoptosis	23	\	\	Postoperative versus preoperative	25 months	Severe blepharoptosis	Efficacy, complication	Single arm
Li 2021[27]	China	Prospective study	Conjoint fascial sheath suspension	53(33/20)	19.87	41.51	CFS versus FMF	3 months	Severe congenital blepharoptosis	Efficacy, complication	Double arm
Qiu 2022[28]	China	Retrospective study	Severe blepharoptosis	32	27	75	Postoperative versus preoperative	8 months	Severe blepharoptosis	MRD1, efficacy, complication, patients’ satisfaction	Single arm
Ahn 2017[29]	South Korea	Cohort study	Mild to moderate blepharoptosis	21	27.5	\	Postoperative versus preoperative	8.9 months	Mild or moderate ptosis	MRD1,efficacy,complication	Single arm
Sang 2021[30]	China	Cohort study	Severe ptosis	110(53/57)	24.85	37.27	CFS versus FM	3 months	Severe ptosis	Efficacy, complication, patients’ satisfaction	Double arm
Xing 2018[16]	China	Retrospective study	Severe congenital ptosis	70	24.19	40	Postoperative versus preoperative	6 months	Severe congenital ptosis	MRD1, patients’ satisfaction	Single arm

*MRD1* marginal reflex distance 1, *CFS* conjoint fascial sheath, *LM* levator muscle, *FMF* frontalis myofascial flap, FM frontalis myofascial

### Meta-analysis

**Fig. 3:**
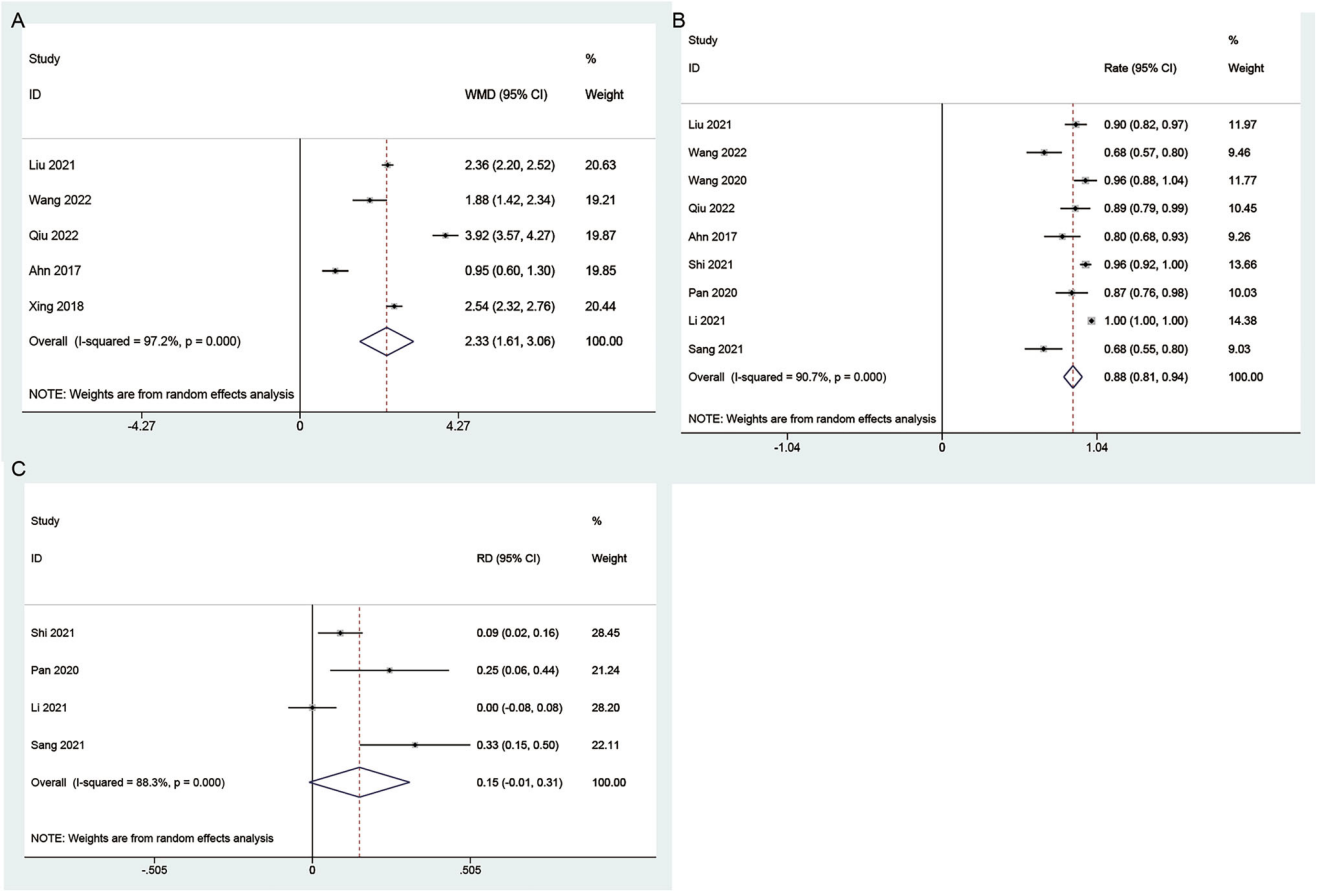
Forest plots of the marginal reflex distance 1 and curative effect in the CFS group. **A** MRD1, **B** curative effect in single arm, **C** curative effect in double arm

*Marginal reflex distance 1 (MRD1)* The meta-analysis revealed a statistically significant improvement in MRD1 in the CFS group compared to the control group (WMD: 2.33; 95% CI 1.61 to 3.06; *I*^2^ = 97.2%, *P* < 0.001) (Fig. 3A). This indicates a substantial enhancement in the position of the upper eyelid in patients undergoing CFS suspension. The results of subgroup analysis based on follow-up time are consistent with the overall analysis results (Fig. S1).

*The curative effect* Nine studies reported outcomes related to the curative effect in blepharoptosis patients. Notably, significant evidence of heterogeneity among the studies was observed (*P* = 0.003, *I*^2^ = 90.7%). The combined proportion of the curative effect in patients undergoing CFS suspension was 0.88 (95% CI = 0.81–0.94) (Fig. 3B). However, the curative effect did not show a significant difference between the CFS group and the control group (RD: 0.15; 95% CI −0.01 to 0.31; *I*^2^ = 88.3%, *P* < 0.001) (Fig. 3C).

**Fig. 4:**
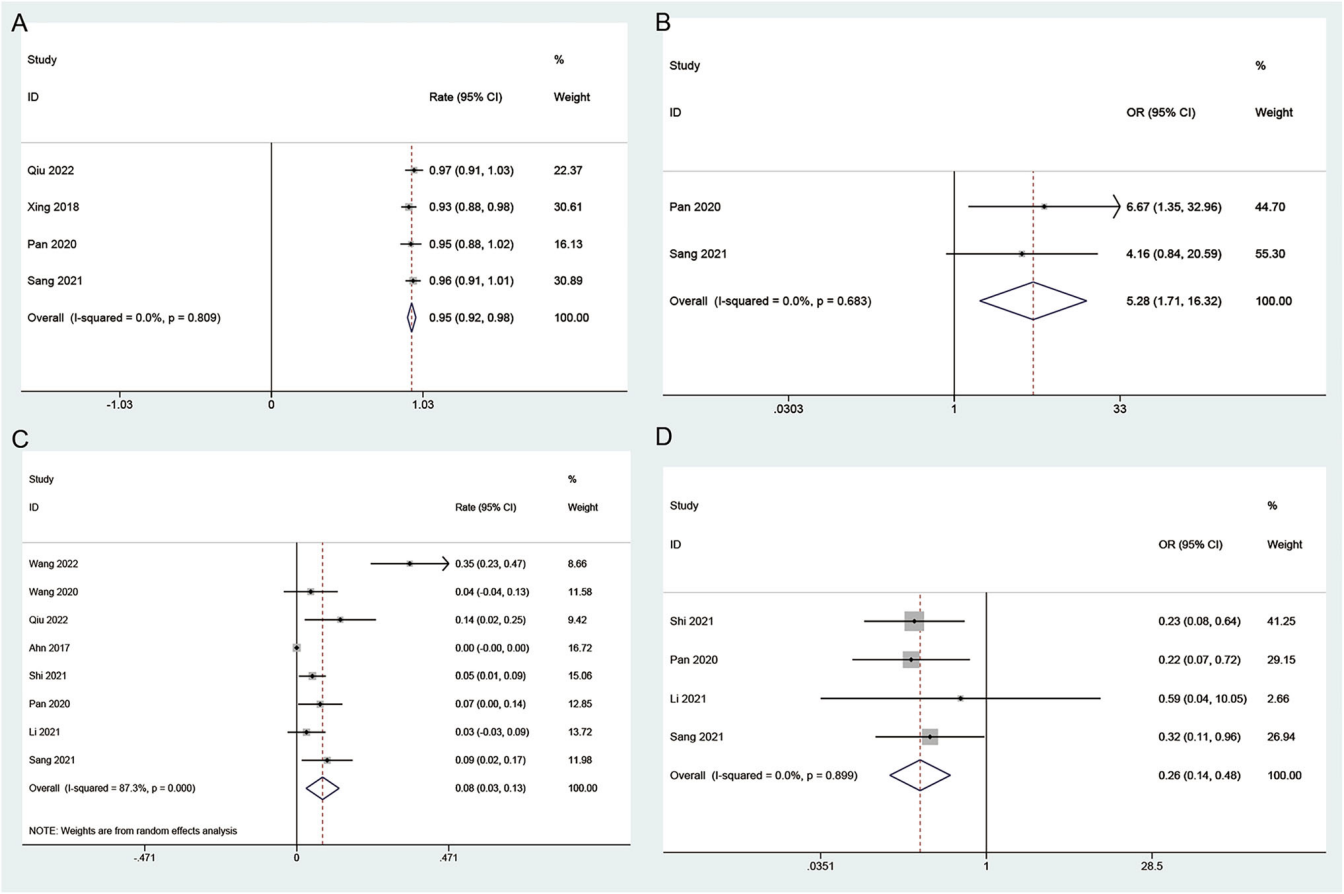
Forest plots of the patient satisfaction and complication rate in the CFS group. **A** Patient satisfaction in single arm, **B** patient satisfaction in double arm, **C** complication rate in single arm, **D** complication rate in double arm

*The patient satisfaction* Four studies reported outcomes related to the patient satisfaction in blepharoptosis patients. Notably, no significant evidence of heterogeneity among the studies was observed (*P* = 0.809, *I*^2^ = 0). The combined proportion of the patient satisfaction in patients undergoing CFS suspension was 0.95 (95% CI = 0.92–0.98) (Fig. 4A). As shown in Fig. S2, the patient satisfaction rate for the follow-up > 6 months was 0.97 (95% CI = 0.91–1.03), while in the case of the follow-up ≤6 months it was 0.95 (95% CI = 0.92–0.98). Analysis of patient satisfaction demonstrated a significant superiority in the CFS group compared to the control group (OR 5.28; 95% CI 1.71 to 16.32; *I*^2^ = 0, *P* = 0.683) (Fig. 4B). Patients undergoing CFS suspension reported a notably higher satisfaction level, emphasizing the positive impact of this intervention on subjective outcomes.

*The complication rate* Eight studies reported outcomes related to the complication rate in blepharoptosis patients. Notably, significant evidence of heterogeneity among the studies was observed (*P* < 0.001, *I*^2^ = 87.3%). The combined proportion of the complication rate in patients undergoing CFS suspension was 0.08 (95% CI = 0.03–0.13) (Fig. 4C). As shown in Fig. S3, the complication rate for the follow-up > 6 months was 0.04 (95% CI = −0.03–0.11), while in the case of the follow-up ≤6 months it was 0.10 (95% CI = 0.03–0.18). The complication rate in the CFS group was significantly lower than that in the control group (OR 0.26; 95% CI 0.14 to 0.48; *I*^2^ = 0, *P* = 0.899) (Fig. 4D). This suggests a favorable safety profile associated with CFS suspension, making it a potentially safer option in comparison to the control interventions.

### Sensitivity Analysis

**Fig. 5:**
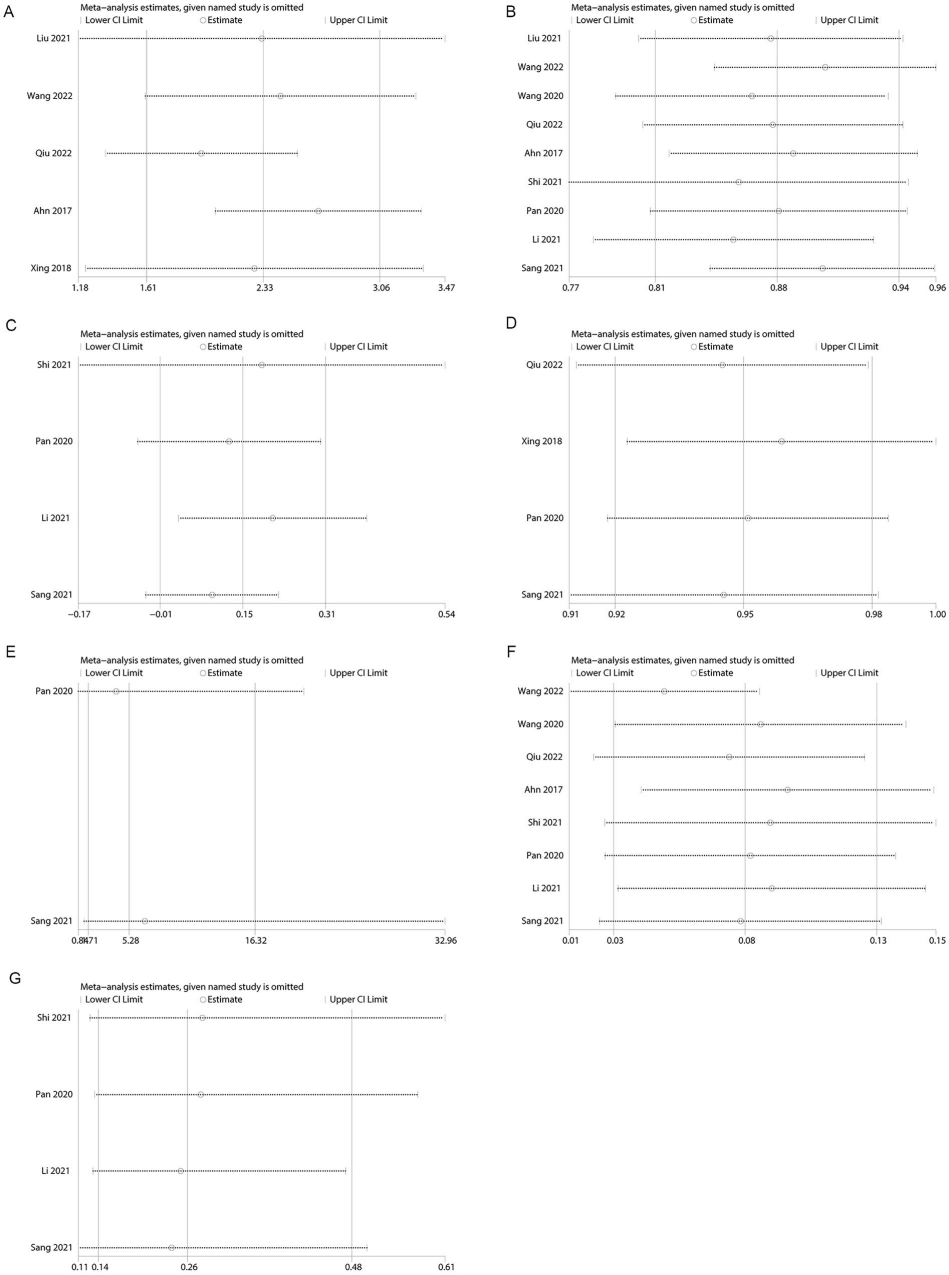
Sensitivity analysis examining the influence of individual studies on pooled results. **A** MRD1, **B** curative effect in single arm, **C** curative effect in double arm, **D** patient satisfaction in single arm, **E** patient satisfaction in double arm, **F** complication rate in single arm, **G** complication rate in double arm

To assess the influence of each individual dataset on the pooled results, sensitivity analyses were carried out by sequentially removing each eligible study. The overall statistical significance remained consistent even when any single study was excluded, indicating the statistical robustness of our results (Fig. 5).

### Publication Bias

**Fig. 6:**
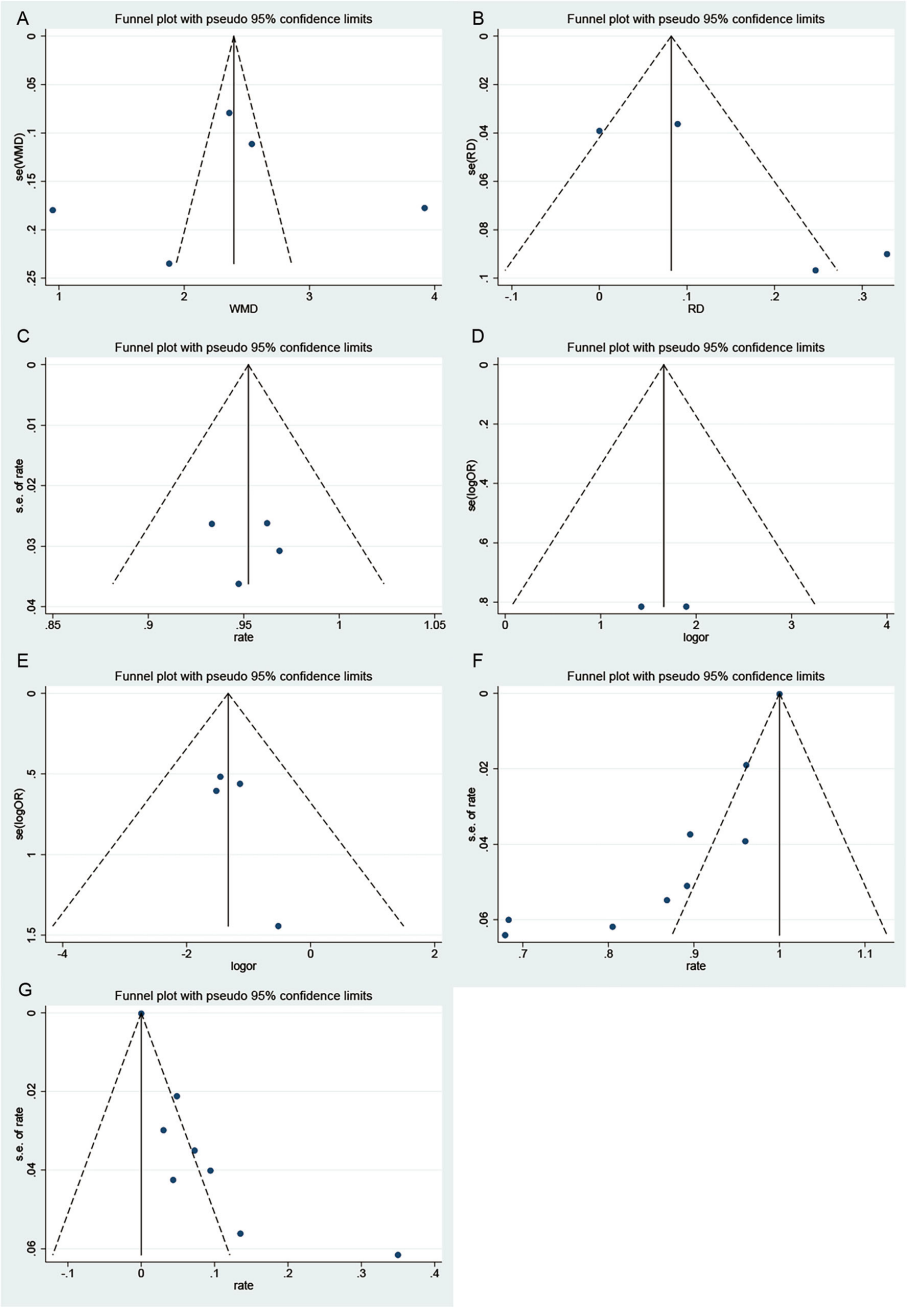
Funnel plot for publication bias. **A** MRD1, **B** curative effect in double arm, **C** patient satisfaction in single arm, **D** patient satisfaction in double arm, **E** complication rate in double arm, **F** curative effect in single arm, **G** complication rate in single arm

Begg’s and Egger’s test were performed to assess publication bias among the studies. No statistical significance of publication bias was observed by Begg’s and Egger’s test to MRD1 (Begg’s test *P* = 0.806; Egger’s test *P* = 0.896) (Fig. 6A), the curative effect in double arm (Begg’s test *P* = 0.734; Egger’s test *P* = 0.158) (Fig. 6B), the patient satisfaction in single arm (Begg’s test *P* = 1.000; Egger’s test *P* = 0.854) (Fig. 6C), the patient satisfaction in double arm (Begg’s test *P* = 1.000) (Fig. 6D), and the complication rate in double arm (Begg’s test *P* = 0.734; Egger’s test *P* = 0.272) (Fig. 6E). However, the *P* value of Begg’s and Egger’s test confirmed the existence of publication bias for the curative effect in single arm (Begg’s test *P* = 0.016; Egger’s test *P* = 0.001) (Fig. 6F) and the complication rate in single arm (Begg’s test *P* = 0.035; Egger’s test *P* = 0.007) (Fig. 6G). The “trim and fill” analysis for the curative effect and complication rate in single arm showed no need for additional literature.

## Discussion

Blepharoptosis, characterized by the drooping of the upper eyelid, presents a complex challenge with both functional and esthetic implications [31]. The exploration of surgical interventions, such as CFS suspension, is essential to refine our approach to managing various types of eyelid ptosis [32]. The systematic review and meta-analysis conducted in this study provide valuable insights into the efficacy and safety of CFS suspension, shedding light on its potential role in addressing this oculoplastic concern.

The primary objective of our study was to assess the efficacy of CFS suspension in the treatment of eyelid ptosis. The meta-analysis revealed a statistically significant improvement in the MRD1 in patients undergoing CFS suspension compared to the control group. This corroborates findings from previous studies [16, 25] that underscore the effectiveness of CFS suspension in achieving objective ptosis correction. This objective improvement in MRD1 indicates a tangible enhancement in the position of the upper eyelid, supporting the efficacy of CFS suspension in achieving the primary goal of ptosis correction. Beyond objective measures, patient-reported outcomes are crucial in evaluating the success of any surgical intervention. Our meta-analysis demonstrated a significantly higher level of patient satisfaction in the CFS group compared to the control group. The odds ratio (OR) of 5.28, with a 95% CI of 1.71 to 16.32, suggests a substantial and clinically relevant improvement in patient satisfaction associated with CFS suspension. The safety of surgical interventions is a paramount concern for clinicians and patients alike. Our study revealed a significantly lower complication rate in the CFS group, aligning with the favorable safety profile reported by Shi et al. [23] and Pan et al. [24]. The OR of 0.26, with a 95% CI of 0.14 to 0.48, indicates a substantial reduction in the likelihood of complications associated with CFS suspension. The absence of heterogeneity in this analysis enhances the internal validity, although variations in complication definitions across studies merit consideration. Despite the observed improvements in MRD1, patient satisfaction, and safety outcomes, our study did not identify a significant difference in the curative effect between the CFS group and the control group. The risk difference (RD) of 0.15, with a 95% CI of −0.01 to 0.31, suggests that, while CFS suspension excels in specific objective and subjective outcomes, the overall curative impact may not be significantly different from alternative interventions.

Positioned between the levator palpebrae muscle and the superior rectus muscle, the CFS represents the connective tissue. It was first characterized as a check ligament of the superior fornix, exerting traction on the tarsal plate to facilitate eyelid movement when the eyeball rotates upward [33]. In 1932, Whitnall introduced the term CFS for the check ligament, a designation that has gained widespread acceptance in later usage. Specifically, he highlighted that this fascial sheath demonstrated a closer connection to the levator muscle than to the superior rectus. In 2002, Holmstrom demonstrated that the pterygoid ligament constituted a connective tissue plate originating from the level of the superior fornix ligament. This ligament was connected to the conjunctival sac, ensuring stability [11]. Utilizing the suspension of this structure to the tarsal plate, he corrected ptosis and achieved positive results [10]. Hwang, in 2008, provided details that the structure had an anteroposterior length of 12.2 ± 2.0 mm (range 8–14 mm), a thickness of 1.1 ± 0.1 mm (range 0.5–1.5 mm), and an equilateral trapezoid shape with a longer anterior base[12]. Located approximately 2.5 ± 0.2 mm (range 2–8 mm) posterior to the superior fornix, it extended from the fascia of the levator and superior rectus posteriorly, 2 mm to the superior fornix anteriorly, and along and beneath the palpebral and bulbar conjunctiva distally. The anatomical insights provided form the basis for correcting ptosis through CFS suspension [34]. As a fibrous tissue that enables the dynamic movement of the upper eyelid, the primary challenge related to CFS suspension—which involves positional adjustment—is similar to the issues faced in any ptosis correction procedure. Specifically, patients may experience a reduction in eyelid dynamic movement after surgery. Given the variety of ptosis correction techniques available and the lack of standardized treatment protocols, a comparative study of these methods is essential.

The findings of our systematic review and meta-analysis have significant implications for clinicians involved in the management of eyelid ptosis. The demonstrated efficacy of CFS suspension in improving objective measures and patient satisfaction positions it as a valuable addition to the armamentarium of ptosis correction techniques. The favorable safety profile further supports its consideration, particularly in cases where minimizing complications is of paramount importance. Clinicians should carefully assess individual patient characteristics, including the severity and etiology of ptosis, to tailor the choice of surgical intervention. While CFS suspension excels in certain outcomes, the absence of a distinct curative effect emphasizes the need for a comprehensive approach to ptosis management. Future studies should delve into specific subtypes of ptosis, considering anatomical variations and underlying causes, to refine our understanding of CFS suspension effectiveness in diverse patient populations.

The nuances of our study warrant consideration of its limitations. Firstly, the meta-analysis included studies with varying designs, patient populations, and surgical techniques, leading to heterogeneity in the results. While efforts were made to account for this through subgroup analyses, inherent differences among studies may have influenced the overall findings. Secondly, the reliance on published data introduces the possibility of publication bias, where studies with positive results are more likely to be published. This bias may result in an overestimation of the positive effects of CFS suspension, affecting the generalizability of the findings. Thirdly, the duration of follow-up varied among the included studies, ranging from short-term to medium-term assessments. This variation may impact the interpretation of long-term outcomes, and the lack of standardized follow-up durations across studies could influence the overall assessment of CFS suspension’s efficacy and safety. Fourth, the study did not provide a detailed analysis of specific subtypes of blepharoptosis or the extent of CFS suspension. Due to the heterogeneity of the disease, the need for CFS extent and prognostic response to CFS suspension may vary among subtypes. However, existing studies primarily focused on patients with severe blepharoptosis, leading to insufficient data for subgroup analysis. Furthermore, the degree of CFS suspension was described as a personalized indicator across all studies, often summarized as “adjusting the upper eyelid height to a satisfactory position.” This limitation reduces the applicability of the findings to specific clinical scenarios. Finally, patient satisfaction, while a crucial outcome measure, is inherently subjective and influenced by various factors such as preoperative expectations and psychological factors. The study did not delve deeply into the nuances of these influences, potentially impacting the comprehensive understanding of patient-reported outcomes.

## Conclusion

In conclusion, our systematic review and meta-analysis provide evidence for the efficacy and safety of CFS suspension in the treatment of various types of blepharoptosis. The observed improvements in mean MRD1, patient satisfaction, and reduced complication rates suggest potential clinical benefits of CFS suspension. Nevertheless, given the heterogeneity of the existing evidence and the limitations of our analysis, these conclusions should be interpreted with caution in clinical practice.

## Supplementary Information

Below is the link to the electronic supplementary material.

Supplementary file1 (PDF 169 KB) Figure S1 Subgroup analysis of MRD1 based on follow-up time.

Supplementary file2 (PDF 168 KB) Figure S2 Subgroup analysis of the patient satisfaction based on follow-up time.

Supplementary file3 (PDF 169 KB) Figure S3 Subgroup analysis of the complication rate based on follow-up time.

Supplementary file4 (DOCX 17 KB) Table S1. Detailed search strategies.

Supplementary file5 (XLSX 11 KB) Table S2. Quality assessment scores for the four non-randomized comparative studies.

## Data Availability

All data generated or analyzed during this study are included in this published article.
