# Supplementary figures and images for: Efficacy and Safety of Conjoint Fascial Sheath (CFS) Suspension in the Treatment of Blepharoptosis: A Systematic Review and Meta-analysis
Source: Aesthetic Plast Surg. 2025 Feb 21;49(15):4427–38. doi: 10.1007/s00266-025-04724-z (PMC12423191; doi:10.1007/s00266-025-04724-z)

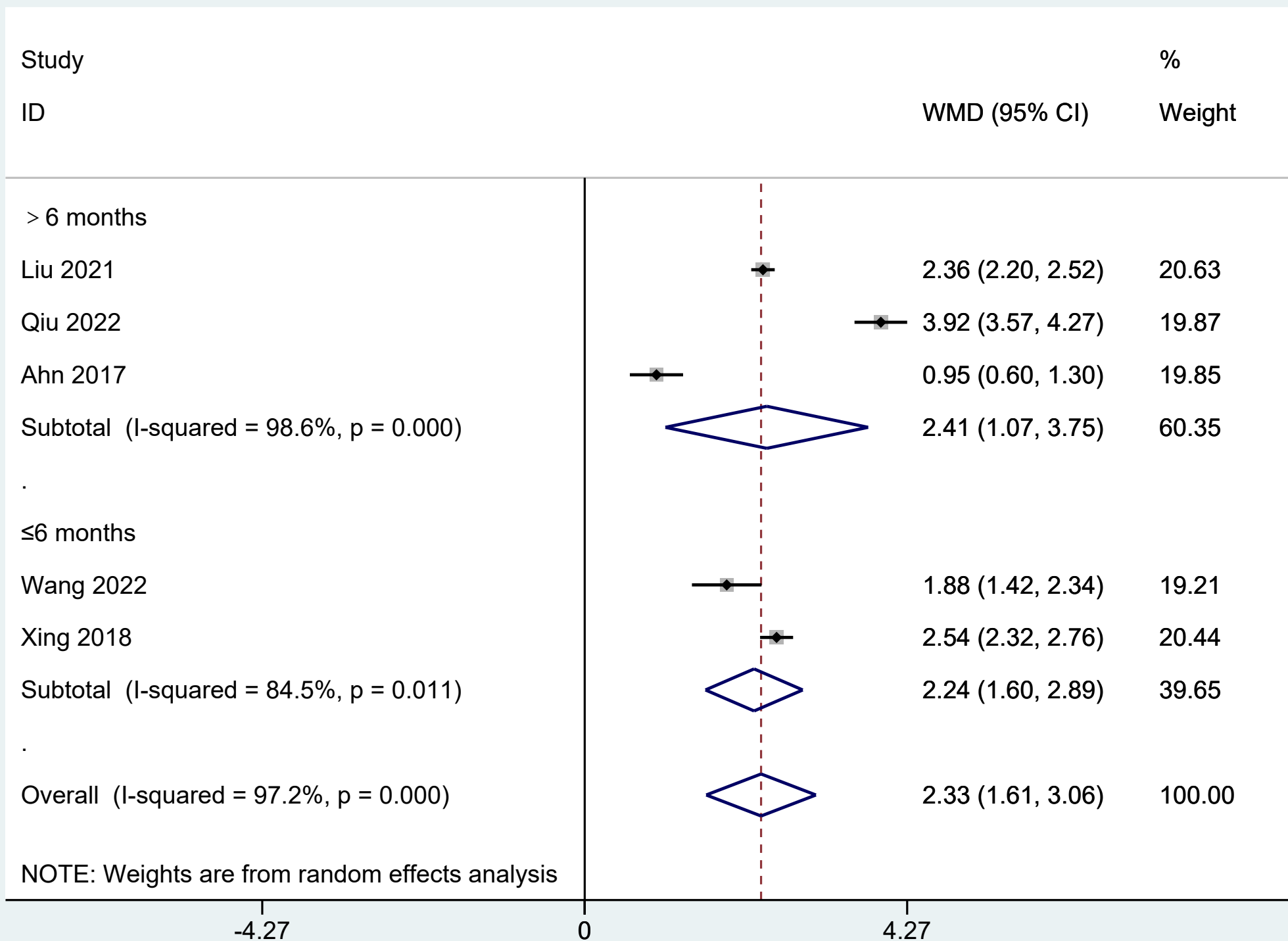

Supplement: Supplementary file 1 — Supplementary file1 (PDF 169 KB) Figure S1 Subgroup analysis of MRD1 based on follow-up time. [file 266_2025_4724_MOESM1_ESM.pdf]

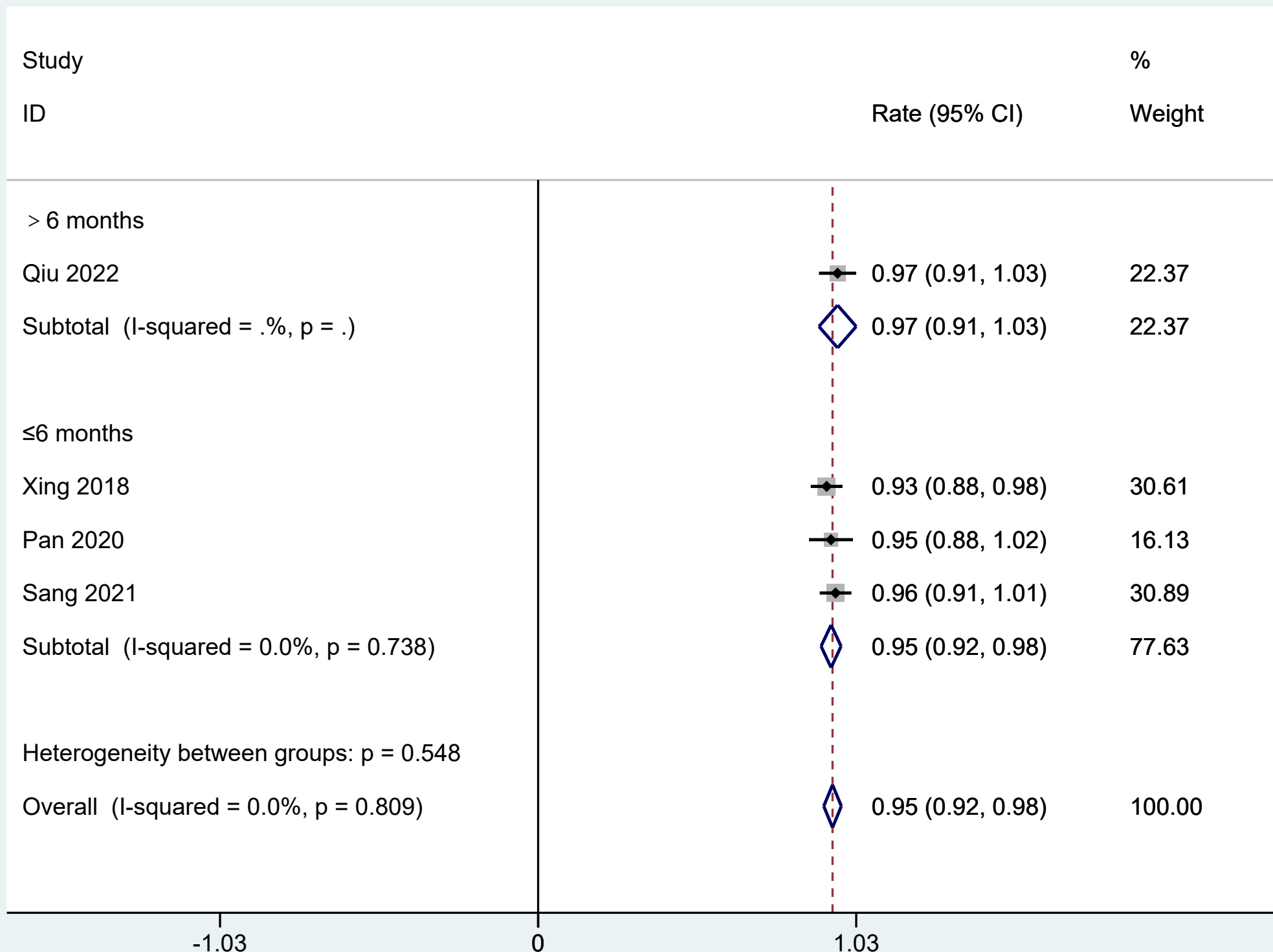

Supplement: Supplementary file 2 — Supplementary file2 (PDF 168 KB) Figure S2 Subgroup analysis of the patient satisfaction based on follow-up time. [file 266_2025_4724_MOESM2_ESM.pdf]

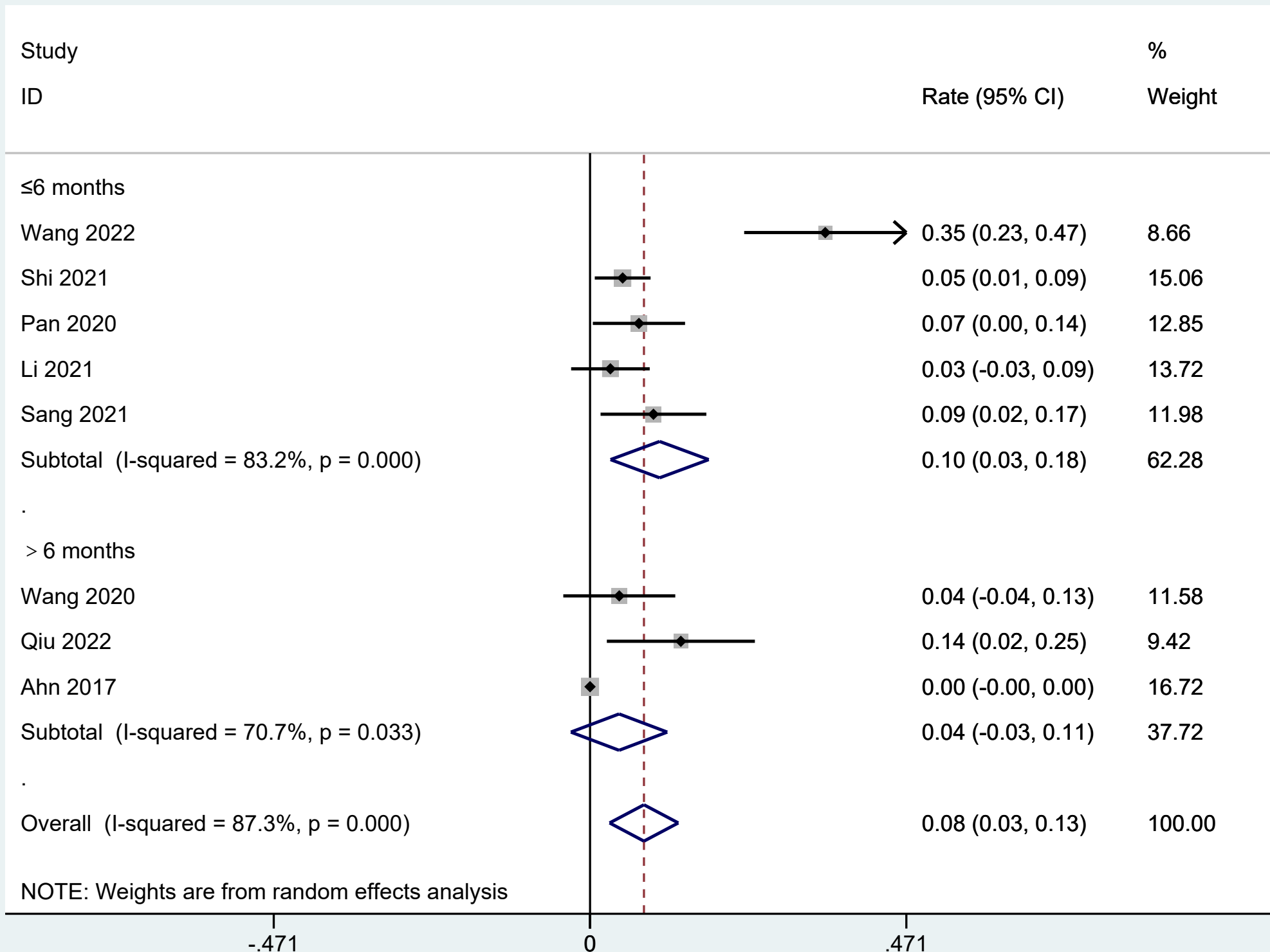

Supplement: Supplementary file 3 — Supplementary file3 (PDF 169 KB) Figure S3 Subgroup analysis of the complication rate based on follow-up time. [file 266_2025_4724_MOESM3_ESM.pdf]
