# Supplementary material for: Efficacy and Safety of Conjoint Fascial Sheath (CFS) Suspension in the Treatment of Blepharoptosis: A Systematic Review and Meta-analysis
Source: Aesthetic Plast Surg. 2025 Feb 21;49(15):4427–38. doi: 10.1007/s00266-025-04724-z (PMC12423191; doi:10.1007/s00266-025-04724-z)
Supplement: Supplementary file 4 — Supplementary file4 (DOCX 17 KB) Table S1. Detailed search strategies. [file 266_2025_4724_MOESM4_ESM.docx]

**Search strategy of PubMed**

| NO. | Search Details | Results |
| --- | --- | --- |
| #5 | #1 AND (#2 OR #3) Filters: Humans | 483 |
| #4 | #1 AND (#2 OR #3) | 544 |
| #3 | ((((((((((((((((((((((Blepharoptoses) OR (Eyelid Ptoses)) OR (Eyelid Ptosis)) OR (blepharo-ptosis)) OR (blepharoptosis)) OR (blepharotosis)) OR (drooping eyelid)) OR (drooping eyelids)) OR (drooping upper eyelid)) OR (drooping upper lid)) OR (droopy eyelid)) OR (droopy eyelids)) OR (dropped eyelids)) OR (eye ptosis)) OR (eye-lid ptosis)) OR (lid ptosis)) OR (ocular ptosis)) OR (palpebral ptosis)) OR (palpebroptosis)) OR (pendulous eyelids)) OR (ptosis)) OR (ptotic eyelid)) OR (ptotic eyelids) | 51,074 |
| #2 | "Blepharoptosis"[Mesh] | 6,348 |
| #1 | (((((conjoint fascial sheath) OR (Fascia suspension)) OR (Levator aponeurosis suspension)) OR (Aponeurosis sling surgery)) OR (Aponeurosis suspension)) OR (Eyelid suspension procedure) | 1,079 |

**Search strategy of EMBASE**

| No. | Query | Results |
| --- | --- | --- |
| #4 | #1 AND (#2 OR #3) | 39 |
| #3 | 'blepharoptoses':ti,ab,kw OR 'eyelid ptoses':ti,ab,kw OR 'eyelid ptosis':ti,ab,kw OR 'blepharo-ptosis':ti,ab,kw OR 'blepharoptosis':ti,ab,kw OR 'blepharotosis':ti,ab,kw OR 'drooping eyelid':ti,ab,kw OR 'drooping eyelids':ti,ab,kw OR 'drooping upper eyelid':ti,ab,kw OR 'drooping upper lid':ti,ab,kw OR 'droopy eyelid':ti,ab,kw OR 'droopy eyelids':ti,ab,kw OR 'dropped eyelids':ti,ab,kw OR 'eye ptosis':ti,ab,kw OR 'eye-lid ptosis':ti,ab,kw OR 'lid ptosis':ti,ab,kw OR 'ocular ptosis':ti,ab,kw OR 'palpebral ptosis':ti,ab,kw OR 'palpebroptosis':ti,ab,kw OR 'pendulous eyelids':ti,ab,kw OR 'ptosis':ti,ab,kw OR 'ptotic eyelid':ti,ab,kw OR 'ptotic eyelids':ti,ab,kw | 18562 |
| #2 | 'ptosis (eyelid)'/exp | 23715 |
| #1 | 'conjoint fascial sheath':ti,ab,kw OR 'fascia suspension':ti,ab,kw OR 'levator aponeurosis suspension':ti,ab,kw OR 'aponeurosis sling surgery':ti,ab,kw OR 'aponeurosis suspension':ti,ab,kw OR 'eyelid suspension procedure':ti,ab,kw | 58 |

**Search strategy of Cochrane Library**

| NO. | Search deatiles | Hits |
| --- | --- | --- |
| #1 | (conjoint fascial sheath):ti,ab,kw OR (Fascia suspension):ti,ab,kw OR (Levator aponeurosis suspension):ti,ab,kw OR (Aponeurosis sling surgery):ti,ab,kw OR (Aponeurosis suspension):ti,ab,kw OR (Eyelid suspension procedure):ti,ab,kw | 55 |
| #2 | MeSH descriptor: [Blepharoptosis] explode all trees | 94 |
| #3 | (Blepharoptoses):ti,ab,kw OR (Eyelid Ptoses):ti,ab,kw OR (Eyelid Ptosis):ti,ab,kw OR (blepharo-ptosis):ti,ab,kw OR (blepharoptosis):ti,ab,kw OR (blepharotosis):ti,ab,kw OR (drooping eyelid):ti,ab,kw OR (drooping eyelids):ti,ab,kw OR (drooping upper eyelid):ti,ab,kw OR (drooping upper lid):ti,ab,kw OR (droopy eyelid):ti,ab,kw OR (droopy eyelids):ti,ab,kw OR (dropped eyelids):ti,ab,kw OR (eye ptosis):ti,ab,kw OR (eye-lid ptosis):ti,ab,kw OR (lid ptosis):ti,ab,kw OR (ocular ptosis):ti,ab,kw OR (palpebral ptosis):ti,ab,kw OR (palpebroptosis):ti,ab,kw OR (pendulous eyelids):ti,ab,kw OR (ptosis):ti,ab,kw OR (ptotic eyelid):ti,ab,kw OR (ptotic eyelids):ti,ab,kw | 531 |
| #4 | #1 AND (#2 OR #3) | 13 |

**Search strategy of web of science**

| NO. | Search deatiles | Hits |
| --- | --- | --- |
| #1 | ((((TS=(conjoint fascial sheath) OR TS=(Fascia suspension)) OR TS=(Levator aponeurosis suspension)) OR TS=(Aponeurosis sling surgery)) OR TS=(Aponeurosis suspension)) OR TS=(Eyelid suspension procedure) | 842 |
| #2 | (((((((((((((((((((((TS=(Blepharoptoses) OR TS=(Eyelid Ptoses)) OR TS=(Eyelid Ptosis)) OR TS=(blepharo-ptosis)) OR TS=(blepharoptosis)) OR TS=(blepharotosis)) OR TS=(drooping eyelid)) OR TS=(drooping eyelids)) OR TS=(drooping upper eyelid)) OR TS=(drooping upper lid)) OR TS=(droopy eyelid)) OR TS=(droopy eyelids)) OR TS=(dropped eyelids)) OR TS=(eye ptosis)) OR TS=(eye-lid ptosis)) OR TS=(lid ptosis)) OR TS=(ocular ptosis)) OR TS=(palpebral ptosis)) OR TS=(palpebroptosis)) OR TS=(pendulous eyelids)) OR TS=(ptosis)) OR TS=(ptotic eyelid)) OR TS=(ptotic eyelids) | 10296 |
| #3 | #2 AND #1 | 338 |
